# The Analysis of ^222^Rn and ^220^Rn Natural Radioactivity for Local Hazard Estimation: The Case Study of Cerveteri (Central Italy)

**DOI:** 10.3390/ijerph20146420

**Published:** 2023-07-20

**Authors:** Nunzia Voltattorni, Andrea Gasparini, Gianfranco Galli

**Affiliations:** Istituto Nazionale di Geofisica e Vulcanologia, Via di Vigna Murata 605, 00143 Rome, Italy; andrea.gasparini@ingv.it (A.G.); gianfranco.galli@ingv.it (G.G.)

**Keywords:** natural radioactivity, indoor radon, thoron, risk hazard, soil gas radon

## Abstract

Radon (^222^Rn) is the second most common cause of lung cancer after smoking. As radon poses a significant risk to human health, radon-affected areas should be identified to ensure people’s awareness of risk and remediation. The primary goal of this research was to investigate the local natural radioactivity (in soils, groundwater, and indoors) because of the presence of tuff outcrops (from middle–lower Pleistocene volcanic activity) that naturally produce radioactive gas radon at Cerveteri (Rome, Central Italy). The results of the radon survey highlighted moderate (>16,000 Bq/m^3^) but localized anomalies in soils in correspondence with a funerary site pertaining to the Etruscan Necropolis of Cerveteri, which extends over a volcanic rock plateau. Indoor radon measurements were performed at several tuff-made dwellings, and the results showed medium-low (<200 Bq/m^3^) values of indoor radon except for some cases exceeding the reference level (>300 Bq/m^3^) recommended by the 2013/59 Euratom Directive. Although no clinical data exist regarding the health effects of thoron (^220^Rn) on humans, the study of ^220^Rn average activity concentration in the soil gas survey reveals new insights for the interpretation of radon sources that can affect dwellings, even taking into account the considerable difference in the half-lives of ^222^Rn and ^220^Rn.

## 1. Introduction

The noble and radioactive gas radon has three isotopes: ^222^Rn (radon, in the decay chain of ^238^U, has a half-life of 3.8 days), ^220^Rn (thoron, in the decay chain of ^232^Th, has a relatively short half-life of 55 s), and ^219^Rn (actinon, in the decay chain of ^235^U). While the first two isotopes are largely studied in geochemical exploration, actinon is completely ignored because of its very short half-life (4 s) and low abundance. 

^222^Rn is one of the main sources of environmental natural radioactivity [1,2] considering that this gas is created during the radioactive alpha decay of radium (^226^Ra) present in rock and soil. Being an inert gas, radon is ubiquitous in indoor and outdoor air. Further, due to its solubility, radon can be present in groundwaters and springs, meaning that drinking waters can be enriched with this gas. Natural ^222^Rn concentrations depend on many factors, including uranium mineralization in the soil [3] and chemical water parameters (pH, temperature, and salinity) that can cause seasonal variations in diluted radon [4]. Furthermore, the presence of fractures and/or faults in the soil can favor the upward motion of radon from bedrocks and buried deposits containing uranium [5]. 

The presence of ^222^Rn in indoor air can be due to the presence of fissures in walls or any other conduits that can facilitate the diffusion of the gas from the ground to closed spaces. Even dissolved radon in groundwaters that are used for domestic purposes can contribute to the indoor accumulation of this dangerous gas [6]. Therefore, it is very important to know the levels of radon in soil gas, the indoor environment, and water (e.g., from private wells) to keep people safe from excessive exposure to radon and, consequently, reduce the lung cancer risk. 

In terms of radiation protection aspects, the indoor ^220^Rn level is attributed to the release of radon from the soil or building materials containing ^232^Th, and they vary depending on the distance from walls or floors [7]. For these reasons, it is difficult to estimate. Furthermore, after thoron decays, its progenies are formed. Most of these are positively charged, and they rapidly capture water molecules, thus forming clusters. They move so quickly in the air that some of them attach to ambient aerosols, while others deposit on the wall, ceiling, floor, and macro-surfaces [8]. Therefore, the distribution of progenies strongly varies and depends on many factors, including deposition velocities and air exchange rates. All these variables affect thoron progeny measurements, and hence the obtained value is only an indicator of its presence and not a quantitative measure of progeny concentrations [9]. By contrast, soil gas ^220^Rn, precisely for its short half-life, can provide significant information on the source of radon gas. 

For this reason, ^220^Rn levels were measured in order to evaluate the superficial fracturing (besides the known faults) that allows for a diffusive radon migration. The present study focused on estimating the radon concentration in soil gas, the indoor environment, and groundwater in an area characterized by natural radioactivity due to both local volcanic outcrops emitting radon and the use of tuffs as building materials. Our primary goal was to raise public awareness of both radon presence and the consequent gas exposure risk [10] since the hazard of this gas is often ignored by local administrators.

## 2. Geological Overview

The investigated area is situated at the Italian Tyrrhenian margin, where large volcanic districts and thick continental crust (less than 25 km) are the main geological characteristics. After the last compressive tectonic phase, which started during the lower Pliocene, an extensional tectonic phase occurred along the Tyrrhenian margin, forming a system of NW–SE-trending basins mainly filled with marine sediments. At the same time, intense volcanic activity began to form the Roman Comagmatic Province, which includes the Sabatini Hills [11] the Tolfa–Cerveteri–Manziana volcanic complex (TVC) [12], and the Vico volcanic deposits.

The studied area belongs to the TVC and is located 45 km north of Rome. The TVC consists of a series of volcanic products (including *Tufi stratificati varicolori di Sacrofano* and *Tufi rossi a scorie nere* [13]) that form an NW–SE-trending hoisted structure. In the southern part of the tuff outcrop, over a volcanic rock plateau of about 100 hectares, there is an important Italian archeological site providing unique and exceptional evidence of the ancient Etruscan civilization, just a few kilometers far from Rome. There are necropolis rich in Etruscan frescoes that faithfully reproduce the daily lives of the disappeared culture. The Banditaccia necropolis, a UNESCO World Heritage since 2004, is by far the finest example of Etruscan funerary architecture, and large parts of this necropolis were dug in the tuff bedrock, either as subterranean grave chambers (hypogea) or as tumuli [14]. 

## 3. ^222^Rn and ^220^Rn Radiological Risk

Radon and its progeny are natural sources of radiation that can affect people’s health, contributing to around 50% of the radiation dose received by the general public [15,16]. According to several epidemiological studies, radon exposure increases the risk of lung cancer since radon progeny deposits on the bronchial epithelium damaging the respiratory system due to irradiation [17,18]. Even cellular DNA can be damaged by the alpha particles emitted by radon and its progeny [19]. The distribution of radon progeny and the breathing rate influence the trachea–bronchial tract [20], where the progeny concentration is deposited, thus affecting the lung’s radiation dose.

Since radon is very soluble (fifteen times more than helium or neon), it is possible to find high concentrations of this gas in waters that circulate through rocks and deposits rich in radium [21] and that can transport dissolved radon to the surface. Therefore, dissolved radon in water can enter dwellings by means of showers, laundry, and drinkable water [16]. Considering the harmful effects of this gas on human health, it is necessary to quantify the radiological hazard of radon from different potential sources: soils, water, and the indoor environment. Since radon is colorless, tasteless, and odorless, the long-term effects of exposure are generally underestimated. Furthermore, even when people are informed about the possibility that their dwellings can have dangerous radon levels and relative health effects, it is unlikely that appropriate remedies are adopted [22].

## 4. Materials and Methods

**Figure 1:**
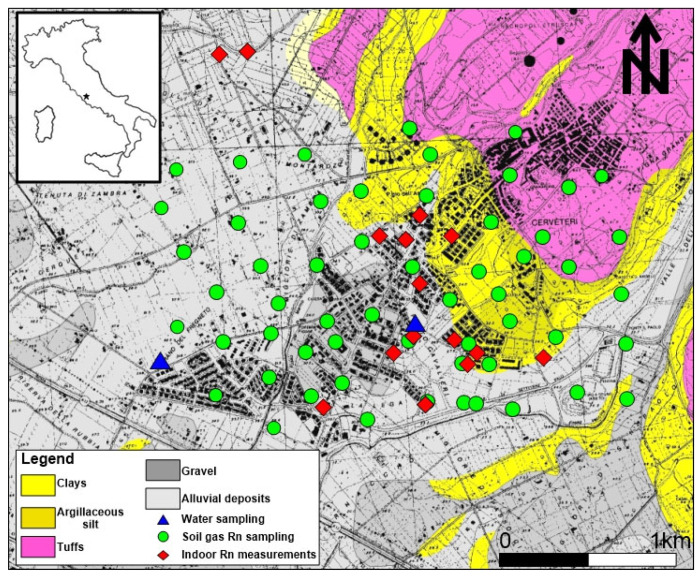
Geological sketch map of the Cerveteri study area (Rome, Central Italy) and radon investigations. Soil gas ^222^Rn and ^220^Rn, indoor radon, and dissolved radon in water are represented by green, red, and blue symbols, respectively.

During the summer of 2017, detailed field surveys were carried out at Cerveteri (Rome, Central Italy), which included indoor, in-water, and in-soil ^222^Rn measurements (Figure 1). 

**Figure 2:**
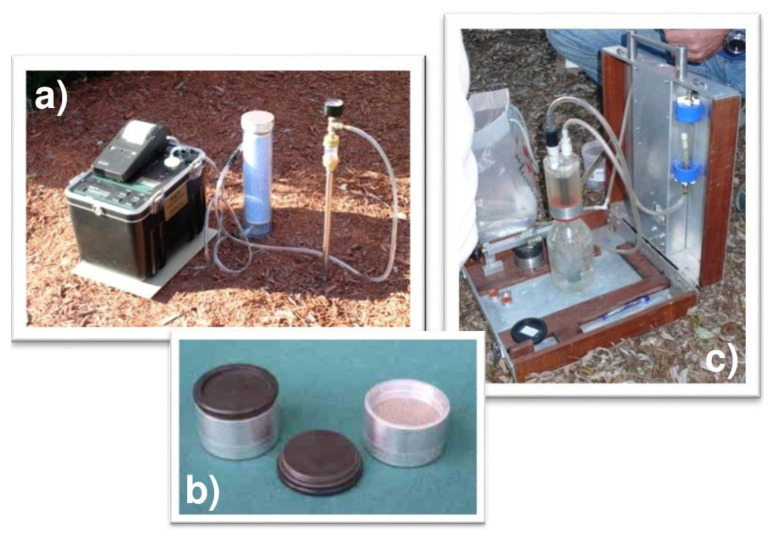
The instruments used for the experiments: (**a**) RAD7 Durridge^®^ alpha spectrometer for ^222^Rn and ^220^Rn measurements in soil; (**b**) activated charcoal collector (ACC); (**c**) dissolved radon collector.

Detailed radon gas surveys were carried out during the dry season (from June to September 2017), consisting of 75 measurements of radon activity, within an area of about 3 km^2^, with a sampling density of about 20 samples/km^2^. Soil gas radon (^222^Rn) concentrations were measured with a portable RAD7 Durridge^®^ certified alpha spectrometer (Figure 2a). Once inside the detection chamber, radon particles produce positively charged ^218^Po ions, which are subsequently collected on the solid-state detector using an electric high-voltage field. In the sniff mode, suitable for fast measurements, only alpha decays from ^218^Po are counted; therefore, temporary radon concentrations were registered in 5 min intervals over a 15 min period (the time necessary for Po and Rn nuclei equilibrium, which is about 5 times the half-life of ^218^Po) by pumping the gas from a steel probe inserted at depth of at least 0.50 m into the soil. An inlet filter and a desiccant trap (drierite) were used to protect the detector from dust and soil moisture (relative humidity should be maintained below 10%). The detection limit was ±500 Bq/m^3^. The calibration and linearity of the employed RAD7s were verified using a radon chamber [23].

All the analytical data obtained were graphically processed to better define geochemical lineaments and anomalies. Statistical analyses were carried out using descriptive statistics (mean, median, variance, standard deviation, skewness, and kurtosis). The geostatistical treatment of gas distribution was performed using a variogram surface analysis to refine the assessment of the spatial continuity of gas concentration anomalies and determine useful parameters for plotting a contour map.

Measurements of indoor radon concentrations were performed in selected private and public dwellings using activated charcoal canisters (ACCs, Figure 2b) and cylindrical aluminum boxes (external diameter 6 cm) that contained activated charcoal. The ACC method is useful for taking measurements simultaneously, and they are reusable after regeneration at 125 °C for at least 10 h since residual radon is expelled. The calibration of activated carbon collectors (ACC) was performed by means of a radon chamber with a volume (V) into which radon activity (RN) was introduced, and the conditions of temperature (T) and relative humidity (RH) varied over time. The ACCs were exposed for a period of 48 h. A calibration factor valid for the specific type of ACCs used was obtained as a function of the parameters indicated above. The typical detection limit is 20 Bq/m^3^ [24].

The ACC passive method involved exposing the canister on the floor of a room for 48 h and keeping windows and doors shut in order to simulate the worst environmental conditions for air circulation. Afterward, the canisters were removed, and the activity of ^222^Rn adsorbed was determined with γ spectrometry. The main γ lines investigated (ROIs) were those emitted in the β decays of ^214^Pb (242, 295, and 352 keV) and ^214^Bi (609 keV). For calculations, it is necessary to (i) select an acquisition time (T) to cumulate counts in the selected ROIs; (ii) measure the background counts (B); (iii) measure the gross counts (G) from each exposed collector; (iv) measure counts (S) from a standard collector (identical in size and material and containing the same amount of activated charcoal) in which a known concentration (A) of ^226^Ra in equilibrium with ^222^Rn is dispersed; (v) calculate a factor (AF) for which the water gain (G) absorbed by the collector and its relation to the specific collector family and exposure time (ET) are assessed using the radon chamber; and (vi) calculate the decay factor (DF) accounting for radon decay from the median exposure time to the median measurement time. Then, these parameters are inserted into the following equation [25]:

Rn (Bq/m^3^) = [(G − B)/T]/[ET × AF(G, ET) × ((S−B)/T)/A) × DF]

In order to measure dissolved ^222^Rn concentrations, water from two private wells was collected in a bottle equipped with a watertight cap provided with an expansion chamber and inserted in a closed circuit (Figure 2c) with a pump and an activated charcoal collector (ACC). The air stripped from the water and aspirated from the expansion chamber was then adsorbed into the ACC [24]. The collectors were analyzed via γ spectrometry using a NaI(Tl) scintillator at the laboratory.

## 5. Results

### 5.1. Soil Gas ^222^Rn and ^220^Rn

The descriptive statistical results of ^222^Rn soil gas levels in the Cerveteri area are reported in Table 1. In an area of about 5 square kilometers, a total of 75 ^220^Rn and ^220^Rn measurements were taken. Radon values ranged from 634 to 51,000 Bq/m^3^, and the mean (13,987 Bq/m^3^) and median (10,000 Bq/m^3^) values, as well as the standard deviation (SD, 11,661), were quite similar, indicating that the distribution of this gas was slightly skewed. The values of skewness (1.35) and kurtosis (2.13) confirmed the almost normal univariate distribution. In the framework of environmental studies, the identification of high anomalous values (outliers) plays a crucial role in the creation and interpretation of contour maps. The normal probability plot (NPP) is a valid statistical approach to display and investigate data variability and skewed distributions. This statistical method enables the identification of threshold values and multiple populations that correspond to relevant processes with their underlying probability density function due to natural processes.

**Figure 3:**
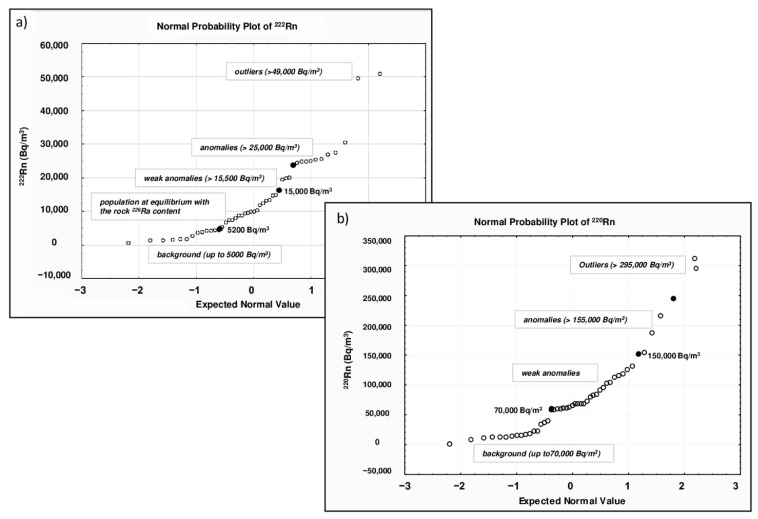
Normal probability plots of ^222^Rn (**a**) and ^220^Rn (**b**). Both plots show some outliers (^222^Rn values > 49,000 Bq/m^3^; ^220^Rn values > 300,000 Bq/m^3^) and different populations (i.e., background and anomalous values) along the probability curve.

The method requires estimating straight line segments (highlighting gaps or inflection points) on a probability curve and then selecting threshold values at the abscissa line [26]. Therefore, it is possible to distinguish populations such as background (natural concentration of soil), anomalous values (values > background), and outlier (extreme data points numerically distant from the rest of the data). Figure 3a shows the normal probability plot for radon values, where we can distinguish some outliers (>49,000 Bq/m^3^) and three sample populations separated by two threshold points: (i) background values up to 5000 Bq/m^3^; (ii) radon values ranging from 5200 to 15,000 Bq/m^3^, characteristic of a population at equilibrium with the ^226^Ra content of the local volcanic rocks [27]; and (iii) weak anomalies (>15,500 Bq/m^3^). The statistical threshold between normal and anomalous values was determined as 25,000 Bq/m^3^. According to Chen and Ford [28], high soil gas concentrations can yield indoor concentrations above the limit established by the 2013/59 Euratom Directive (300 Bq/m^3^) [29]. Only a few samples in our soil gas dataset had values so high to potentially result in high indoor radon concentrations, but those samples were collected from the tuff outcrop, where there is the Banditaccia necropolis and no dwellings. The descriptive statistical results of ^220^Rn soil gas levels in the Cerveteri area are reported in Table 1. Thoron values ranged from 848 to 312,000 Bq/m^3^. The mean (76,616 Bq/m^3^) and median (65,200 Bq/m^3^) values, as well as the standard deviation (SD, 65,533), were almost identical, indicating that the distribution of this gas was slightly skewed. The values of skewness (1.6) and kurtosis (3.1) confirmed the almost normal univariate distribution. The presence of outliers (high anomalous values) was determined using the mean of the NPP graph (Figure 3b), where we can distinguish three sample populations separated by two threshold values: (i) background values up to 70,000 Bq/m^3^; (ii) weak anomalous values ranging from 70,000 to 150,000 Bq/m^3^; (iii) anomalies (>150,000 Bq/m^3^). The anomaly threshold is at 155,000 Bq/m^3^.

### 5.2. Indoor ^222^Rn

A total of 24 charcoal canisters were arranged on the floor of 24 private locations where a room could be dedicated to indoor radon measurements. Canisters should be placed at the lower level of a building like cellars (i.e., storerooms) or basements (hobby rooms or workrooms), but this was not always possible because of the different types of available dwellings. For this reason, canisters were placed in seven cellars, seven basements, five ground floors, and five first floors. Every location was built with tuff and without crawl space or any modern aeration system for radon abatement. Table 2 shows the results of indoor radon measurements: Values ranged from 35 to 1144 Bq/m^3^. SD and mean values of basements were almost similar, suggesting an almost normal distribution (Gaussian distribution). However, the values of the coefficient of variation (CV = SD/mean) suggest that data variability for cellars, ground floors, and first floors (CV = 0.5, 0.6, and 0.5, respectively) but not for basements (CV = 1.1).

**Figure 4:**
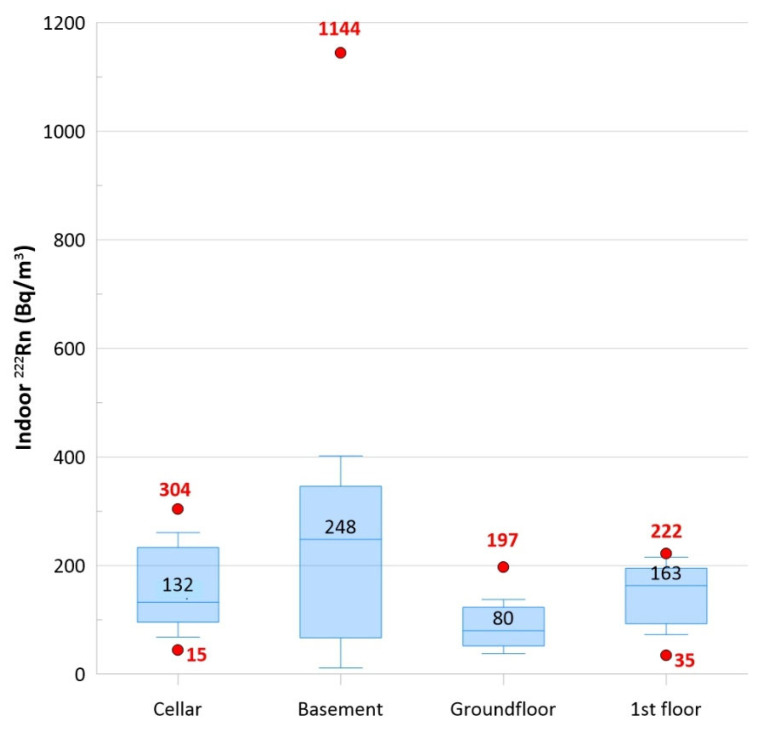
The box plots show a comparison of indoor radon values from the four monitored levels (cellars, basements, ground floors, and first floors). The boxes were plotted using lower and upper quartiles (edges of the box), the median (line and number at the center of the box), whiskers (defined by the IQR factor), and outliers (red dots and relative red numbers).

Figure 4 shows a comparison of indoor radon values from the four monitored levels. The box plots were drawn using lower and upper quartiles (box edges), the median (the line at the center of the box), whiskers (defined by the IQR factor), and outliers. This graphical representation of data provides a brief and concise summary of the variability of values, as shown with the median values estimated for the different levels: basements had the highest values, as expected. 

### 5.3. Dissolved ^222^Rn in Water

Dissolved radon in water can be a serious problem, in terms of radiation dose, when groundwater is used for public services (e.g., food and domestic uses, sanitary fittings, and private wells). Radon dissolved in water may enter indoor air through de-emanation when the water is used, and its contribution to indoor Rn concentrations mainly depends on the dissolved radon concentration, the amount of water used, and the air exchange rate. The ratio of concentrations in the air and water was set at 10^−4^ [16] (i.e., a dissolved concentration of 100 Bq/L would cause an increase of 10 Bq/m^3^). Although this contribution to indoor radon is normally negligible, radon degassing from the water should not be underestimated. 

Unfortunately, during the study period, only two private wells were investigated, both with very low radon concentrations, that is, <100 Bq/L (the reference radon level of drinking water according to the 2013/59 Euratom Directive). However, these low values are comparable with the results of a previous study by Cinti et al. [30] in the peri-Tyrrhenian sector of Central Italy, where there are sedimentary rock aquifers that generally have low dissolved Rn values (median value 6.9 Bq/L) probably due to low levels of U and Ra within the sedimentary deposits. 

## 6. Discussion

**Figure 5:**
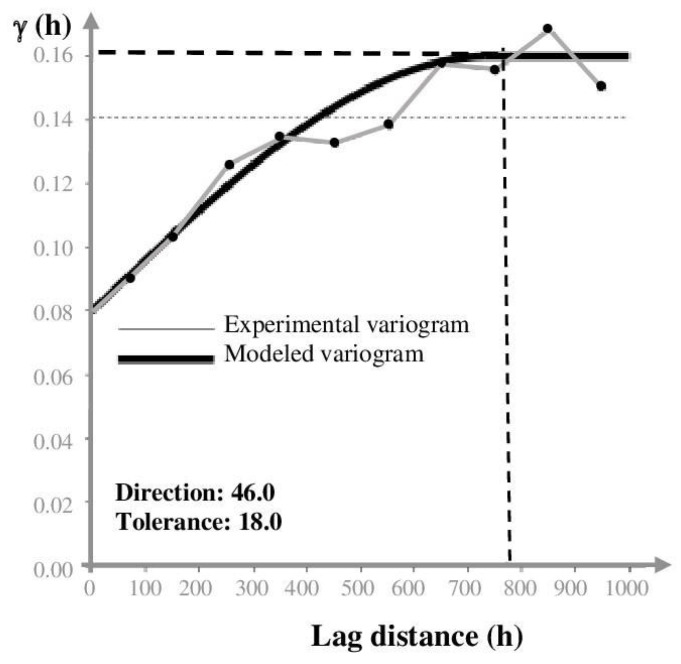
Experimental variogram and the relative fitting model for radon data. The fitted model is a spherical function for which at a distance greater than 700 m, sample pairs will no longer be autocorrelated, and thus the variogram reached the asymptotic threshold value of 0.16 γ (horizontal dashed line). Vertical dashed line indicates the sill, that is the variogram value where the variogram function flatten off at increasing distance.

A variogram model was built using radon data to quantitatively assess the spatial continuity of the values. An experimental variogram enables the comprehension of the geometry and continuity of one variable and can provide significant information on numerical model estimations. Variogram models estimate the experimental data, geological interpretation, and analog information [31]. Figure 5 shows the experimental variogram and the relative radon fitting model determined along the axes of anisotropy (45°) and an angular tolerance of 22°. The x-axis represents the lag distance (distances between pairs at which the variogram is calculated), and the y-axis refers to the equation for computing the variogram and depends on the variance of the variable as a function of the lag distance. 

**Figure 6:**
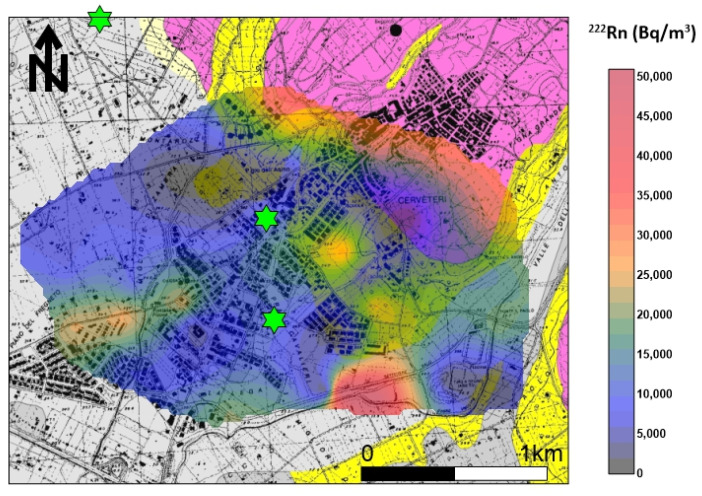
^222^Rn soil gas concentration distribution. The maximum anisotropy orientation (NW–SE) is parallel to the tuff outcrop where the Banditaccia necropolis is located. Other spot anomalies (values > 25,000 Bq/m^3^) were found in the southeastern sector of the investigated area, where small tuff outcrops or Etruscan ruins are present. Green stars indicate indoor ^222^Rn values > 300 Bq/m^3^.

The appropriate model was a spherical function (Sph) in which sample pairs were autocorrelated up to the distance of 700 m; after that, the variogram reached the asymptotic threshold value of 0.16 γ. The nugget variance, representing the threshold value, was 0.08 γ. The variogram model and the calculated values were fundamental in the Kriging algorithm to perform the most accurate evaluation of the radon concentration’s contour map, especially in areas without sampling. Figure 6 shows the distribution of soil gas radon concentrations, which were very variable in the investigated area. The mean activity of the radon was about 14,000 Bq/m^3^, a value that is considered not harmful to human health. The maximum anisotropy orientation (NW–SE) is parallel to the tuff outcrop where the Banditaccia necropolis is located. Other spot anomalies (values > 25,000 Bq/m^3^) were found in the southeastern sector of the investigated area, where small tuff outcrops or Etruscan ruins are present. Furthermore, an elongated anomaly was found in the southwestern sector that is apparently not subject to either known faults or tuff outcrops. According to Torelli [32], there are several necropoles (behind the well-known Banditaccia) in the investigated area, and many of them are underground (hypogeal necropoles). This would explain the anomalous values in the southern sector of the studied area, although more detailed investigation (e.g., geoelectrical surveys) are needed to confirm whether there is an association between radon levels and buried necropoles.

**Figure 7:**
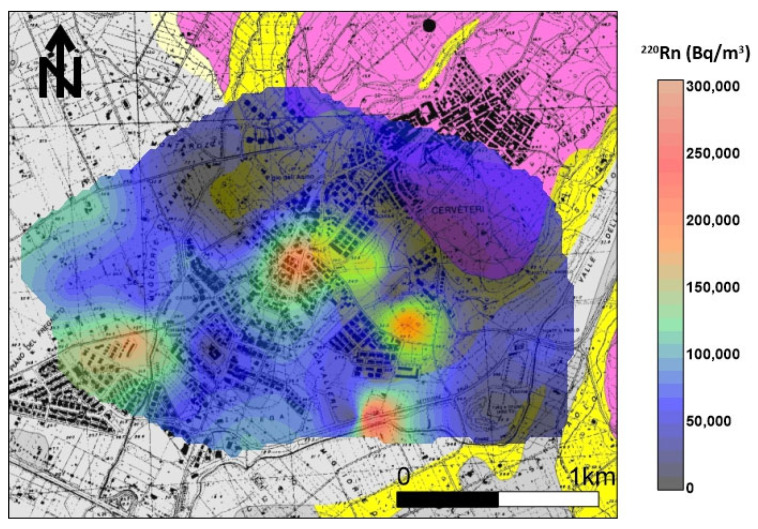
^220^Rn soil gas concentration distribution. The maximum anisotropy orientation (NE–SW) is orthogonal to the tuff outcrop. Other spot anomalies (values > 90,000 Bq/m^3^) were found in the southeastern sector of the investigated area in line with ^222^Rn anomalies and where small tuff outcrops or Etruscan ruins are present.

The distribution of soil gas ^220^Rn shows (Figure 7) that the maximum anisotropy orientation (NE–SW) is orthogonal to the tuff outcrop. Other spot anomalies (values > 90,000 Bq/m^3^) were found in the southeastern sector of the investigated area in line with ^222^Rn anomalies and where small tuff outcrops or Etruscan ruins are present.

**Figure 8:**
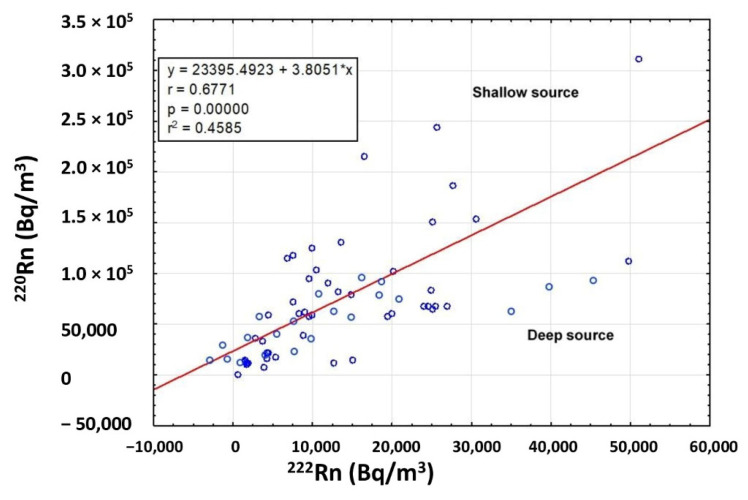
Correlation between ^222^Rn and ^220^Rn activity. The graph shows a good correlation (Pearson coefficient r = 0.6771) between the measured radon and thoron concentrations, indicating a mixing of deep and shallow gas sources.

The short half-life of ^220^Rn (55 s) is long enough to suggest the primary source (U or Th) of radon anomaly. Therefore, the presence of mineralization close to the surface can cause high ^220^Rn levels due to the rapid transport of this gas. The calculation of the ^220^Rn/^222^Rn activity ratio allows us to investigate the actual permeability of the gas origin. A good correlation was observed (Pearson coefficient r = 0.6771) between the radon and thoron activities (Figure 8), indicating and confirming a mixing of deep (^222^Rn) and shallow (^220^Rn) gas sources [33]. According to Yang [34], thoron values associated with low radon values suggest a shallow gas source since the origin of thoron is almost entirely from diffusion from the top few centimeters of soil where this gas is emitted as a decay product of ^232^Th contained in soils and rocks. Furthermore, microfractures in the soil facilitate the migration and presence of this gas at the surface.

Since thoron is more sensitive to the shallow fracturing system, a high ^220^Rn/^222^Rn activity ratio suggests a high level of ground permeability, which enhances the thoron escape toward the surface.

**Figure 9:**
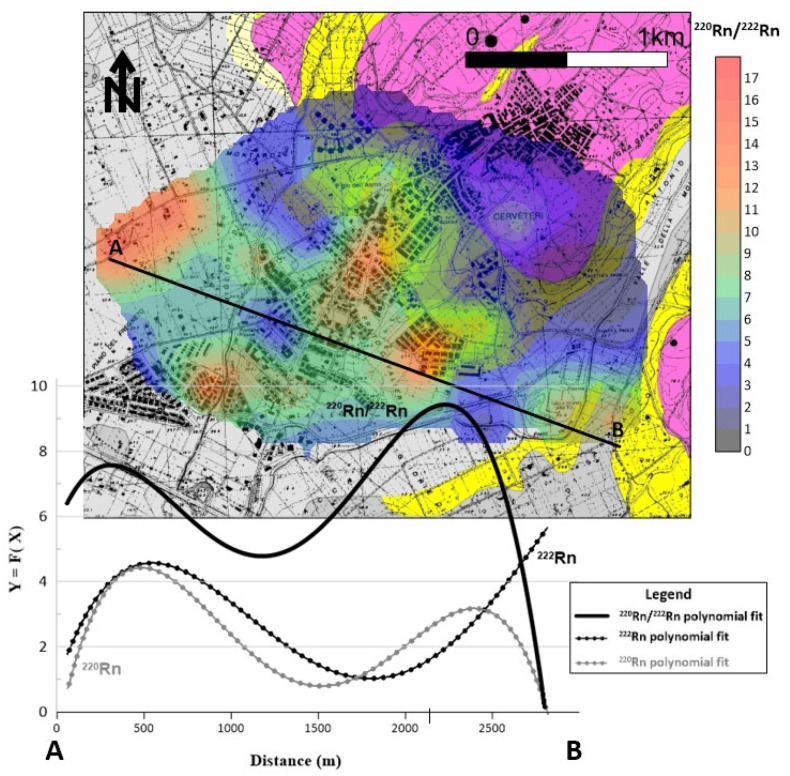
The third-order polynomial regression of ^220^Rn/^222^Rn activity ratios is plotted on A-B profile. The high activity ratios found in both the northwestern and central part of the investigated area suggest the presence of discontinuities (faults/fractures as well as hypogeal necropoles) not visible at the surface.

Figure 9 shows the third-order polynomial regression of the ^220^Rn/^222^Rn activity ratios plotted along the A-B profile. The highest values of the regression curve are in the northwestern and central part of the studied area, where there is a correspondence between radon and thoron values. This correlation reflects the common physical factors affecting the transport of both, such as diffusivity, moisture, and the presence of gas sources and migration pathways not visible at the surface. The small areas where the ^220^Rn–^222^Rn correlation exists are characterized by the presence of argillaceous and alluvial deposits (without tuff outcrops). Therefore, it is reasonable to assume that the high activity ratios observed in these areas would be due to local hidden discontinuities (faults/fractures) and/or to unknown hypogeal necropoles.

Many studies [35,36,37] suggest a direct correlation between indoor radon and soil gas radon levels. The highest soil gas radon values were found in tuff outcrops where, unfortunately, no indoor radon measurements were taken because of the presence of Banditaccia necropolis. On the other hand, the highest indoor radon values were found in dwellings located on alluvial deposits, where soil gas radon concentrations were very low (<15,000 Bq/m^3^). Most parts of ACC were placed in dwellings located on argillaceous and alluvial deposits, which are well known for acting as barriers to the migration of gas and other substances of geological eras, as evidenced by the clay-capped deposits of hydrocarbons over the entire planet [38]. Only three samples had high indoor radon values (>300 Bq/m^3^), and they were measured in one cellar and two basements located on alluvial deposits, where soil gas radon concentrations were very low (Figure 6). The cause of high indoor radon levels may be attributed to the construction features of the dwellings and the materials used: According to Righi and Bruzzi [39], Central Italy is characterized by volcanic rocks (i.e., tuffs), which are used as building materials and are natural sources of radioactive emissions. Therefore, the natural radioactivity in tuff bricks plays an important role in indoor radon accumulation that should be determined for hazard estimation and land use planning. The results of indoor radon measurements confirm that dwellings built over volcanic tuffs can undergo harmful radon gas accumulation. An exception is the results from the first-floor level: Only two samples had values < 100 Bq/m^3^, while the remaining exceeded 160 Bq/m^3^, with one sample reaching 222 Bq/m^3^. However, these high values are below the radon threshold admitted for indoor radon exposure (<300 Bq/m^3^) by the 2013/59 Euratom Directive [29]. A possible explanation for the high values observed for the first floors is the “chimney effect” caused by temperature differences (between outside and inside a house), leading to a variation in radon concentrations due to airflow, especially in the winter season. All indoor measurements were taken in winter, during which, notoriously, maximum values of radon concentration are observed. This is because windows are usually closed during winter, causing scarce aeration in the indoor environment. Furthermore, winter variations in barometric pressure can have a positive effect on radon concentrations: an increase in barometric pressure causes an increase in radon concentration, especially if accompanied by windstorms and rain [40]. 

All the investigated cellars were used as stock rooms with no heating system, and, consequently, natural ventilation had a significant effect on the indoor radon concentration. In fact, when the ventilation rate in the cellar increased, the cellar became less depressurized, leading to a decrease in radon permeation into the cellar. Therefore, the concentration in the cellar decreased due to stronger dilution and the reduced level of the contaminant permeated into the cellar [41]. 

## 7. Conclusions

The multiple and simultaneous investigations conducted in this work proved to be a better approach for the accurate identification of radon natural radioactivity in terms of potential health risks due to exposure to this dangerous gas.

A detailed radon survey (with in-soil, indoor, and in-water measurements) was conducted in the Cerveteri area (50 km north of Rome, Central Italy), within a densely populated area, to identify the radon source and the main causes influencing the distribution of this gas. The contour maps of both ^222^Rn and ^220^Rn soil gas distribution were analyzed using variogram models established with the Kriging algorithm. In particular, the study of radon distribution highlighted an NW–SE anisotropy pattern, which was correlated to the limit of tuff outcrops, naturally producing radon. Lithology, therefore, is the main cause of the ^222^Rn distribution in soil, but other causes (e.g., the presence of necropolis), depending on location, were explored to explain the radon variation.

Exposure to indoor radon can pose a risk to human health, and the achieved results demonstrate that the risk is consistent in cellars/basements and, to a lesser extent, first floors of dwellings. It is worth noting that this gas accumulates in ground contact and poorly ventilated places. The absence of correlation between soil gas and indoor ^222^Rn levels highlights the importance of building materials, often the main reason for indoor radon levels in the study area. 

Taken together, the obtained results show that the presence of degassing soils and building materials strongly contribute to the indoor Rn distribution, which should be determined for hazard estimation and land use planning. It is firmly recommended that local authorities and citizens be informed about the potential health risk of this toxic gas, and its origin, characteristics, and spatial distribution. Even though few houses had high indoor radon values above the radon reference level allowed for indoor radon exposure (300 Bq/m^3^) by the 2013/59 Euratom Directive, seasonal variations should be considered in order to calculate the annual effective dose equivalent. Meanwhile, more ventilated entry areas and the presence of air-conditioning with a regulated exchange of air would facilitate the radon escape from “contaminated” houses.

## Figures and Tables

**Table 1:** Descriptive statistical results of ^222^Rn and ^220^Rn soil gas levels in the studied Cerveteri area.

	222Rn (Bq/m^3^)	220Rn (Bq/m^3^)
Samples	75	75
Min value	634	848
Max value	51,000	312,000
Mean	13,987	76,616
Median	10,000	65,200
Anomaly threshold	17,000	150,000
Variance	1.36 × 108	4.29 × 109
Standard deviation	11,661	65,533
Skewness	1.4	1.6
Kurtosis	2.1	3.1
Kolmogorov–Smirnov	0.2	0.2

**Table 2:** Descriptive statistical results of indoor radon levels in the studied Cerveteri area. N: number of samples, LQ: lower quartile, UQ: upper quartile, SD: standard deviation.

Indoor Rn (Bq/m^3^)	N	Min	Max	Median	Mean	CV	LQ	UQ	SD
Cellar	7	45	304	149	157	0.5	101	227	89
1st floor	5	35	222	164	142	0.5	79	202	77
Ground floor	5	43	197	80	99	0.6	50	142	63
Basement	7	49	1144	248	327	1.1	78	341	380

## Data Availability

The data cannot be made publicly available upon publication because they contain sensitive personal information. The data that support the findings of this study are available upon reasonable request from the authors.
